# Vitamin D in the elderly: the phil-rouge in preventing bone, muscle and adipose deterioration?

**DOI:** 10.20945/2359-4292-2025-0281

**Published:** 2025-11-24

**Authors:** Luigi di Filippo, Umberto Terenzi, Andrea Giustina

**Affiliations:** 1 Institute of Endocrine and Metabolic Sciences, Università Vita-Salute San Raffaele, IRCCS Ospedale San Raffaele, Milano, Italia

**Keywords:** Vitamin D, elderly, osteoporosis, osteosarcopenic obesity, COVID-19

## Abstract

The pleiotropic role of vitamin D in human health has been implicated in modulating bone metabolism and other several extraskeletal areas, including muscle and adipose tissues regulation, and in influencing general and systemic outcomes. In the elderly, vitamin D deficiency is considered as an emerging public health issue affecting 40%-70% of older adults worldwide with higher rates occurring in institutionalized individuals or patients with multiple chronic comorbidities. The pathophysiology of vitamin D deficiency in the elderly is multifactorial and includes age-related reduced skin synthesis, limited sun exposure, declined renal and liver function, and long-term use of interfering medications. Given its pleiotropic effects, vitamin D deficiency in the elderly has been consistently associated with progressive bone deterioration and muscle and adipose dysfunctions, concurring to the occurrence of the osteosarcopenic obese phenotype. This multifaced deleterious scenario is strongly correlated with an increasing risk of fragility fractures, falls, functional and metabolic decline, all of which contribute to higher morbidity and mortality. Early diagnosis and screening with individualized criteria, targeted and personalized strategies for supplementation, and structured follow-up monitoring are required to reduce the clinically significant impact of vitamin D deficiency in this highly vulnerable population.

## VITAMIN D DEFICIENCY IN THE ELDERLY: MECHANISMS AND EPIDEMIOLOGY

### Pathophysiological mechanisms

Vitamin D is a fat-soluble prohormone that plays central roles in calcium-phosphorus and bone metabolisms, and in several extra-skeletal conditions influencing muscle and adipose health, and cellular growth (^1^). In humans, vitamin D is mostly synthesized endogenously by the skin after the exposure to ultraviolet B (UVB 290-315 nm) radiation, and, in a minor part, it can be also obtained exogenously through foods ingestion (^1^,^2^). The two main primary forms of vitamin D are cholecalciferol (vitamin D3) and ergocalciferol (vitamin D2). Cholecalciferol is the biologically most common form synthesized in the skin and present in animal-based foods such as fatty fish, egg yolk, and dairy products. Instead, ergocalciferol is mostly derived from plants and fungi (^1^). Cholecalciferol and ergocalciferol differs in structural conformation for the presence of an additional carbon double-bond and a methyl group in the side chain of vitamin D2, and in available sources for humans since vitamin D3 is primarily synthesised in the skin and, in contrast, vitamin D2 is exclusively obtained from exogenous sources (^3^). In humans, vitamin D2 and D3 were considered equally biologically active for several decades, but current knowledge indicates that the efficacy of vitamin D2 is less than one-third of vitamin D3 (^3^).

The cutaneous skin synthesis is the primary source of vitamin D3 for most individuals, and it is influenced by aging, season, latitude, skin pigmentation, and sun exposure habits. In subjects with limited UVB exposure, such as older adults, individuals living at high latitudes, or those with occupational or cultural sun restrictions, the vitamin D dietary intake and supple mentation are critical sources of this hormone (^4^). Once produced by the skin or introduced from foods sources, circulating vitamin D is biologically inactive until it is processed by a two-step hydroxylation process occurring the first-one in the liver and the second-one in the kidneys to finally constitute the biologically active form responsible for most of the vitamin D systemic effects.

Aging significantly alters vitamin D metabolism impairing each process involved in its synthesis and activation by reducing cutaneous synthesis, impairing liver and renal functions, and also by altering vitamin D bioavailability in circulation (^4^). These changes occurring in the elderly population can be combined with additional behavioural and environmental factors, as dietary restriction and prolonged institutionalization, further impairing vitamin D metabolism and contributing to the very high prevalence of vitamin D deficiency commonly present in older subjects (^5^).

One of the most detrimental effect of aging on vitamin D metabolism is the progressive decline in skin synthesis of vitamin D3. This decline is mainly related to a gradual reduction in epidermal 7-dehydrocholesterol levels, the precursor molecule required for vitamin D3 synthesis. It is well demonstrated that at the age of 70-80 years, 7-dehydrocholesterol cutaneous decreases by 50%-75% as compared to younger population, significantly impairing the cutaneous ability to synthesize vitamin D3 (^4^). Indeed, recent studies estimate that vitamin D3 cutaneous synthesis declines by approximately 13% per decade, and older individuals effectively produce only about one-half of the vitamin D3 that younger adults (^6^). Moreover, besides the marked reduction in vitamin D3 cutaneous synthesis, additional several behavioural changes typically occurring in the elderly are known to negatively influence vitamin D metabolism. Spending less time outdoors, wearing more protective clothing, and avoiding sun exposure due to concerns about skin cancer and photoaging are known to reduce the skin sun exposure and the associated vitamin D synthesis (^4^). Institutionalized subjects and individuals with mobility impairments are recognized to be particularly vulnerable as they experience minimal UVB exposure (^7^). In addition, in respect to western population, geographical and seasonal factors further influence vitamin D synthesis since in regions above 37° latitude, as for most of Europe and North America countries, UVB radiation is known to be markedly insufficient during winter months to stimulate vitamin D cutaneous production (^4^).

Organ dysfunctions, especially involving liver and kidneys, and age-related comorbidities, can be associated to a further disruption in vitamin D metabolism. The first hydroxylation step necessary for the activation of vitamin D metabolites occurs in the liver where the enzyme vitamin D-25-hydroxylase (CYP2R1) converts both cholecalciferol and ergocalciferol into 25-hydroxyvitamin D [25(OH) vitamin D] (also named calcifediol), the main circulating form. There is also a unique additional activation pathway for vitamin D2 promoted by hepatic 24-hydroxylase (CYP24A1) leading to 24(OH) vitamin D2 synthesis that can undergo to further activation (^3^). Chronic liver disease, as in case of cirrhosis and non-alcoholic fatty liver disease (NAFLD), are known to profoundly impair the CYP2R1 activity reducing 25(OH) vitamin D synthesis (^8^,^9^). While these detrimental effects of chronic liver diseases are well-known, evidence suggests that elderly itself can affect hepatic vitamin D metabolism even in the absence of overt liver pathological conditions (^5^,^6^). With increasing age, the hepatic CYP2R1 activity progressively declines, and liver volume and hepatic blood flow decrease by 0.3%-1.5% per year after the age of 40 years (^10^). Moreover, older age is also associated with progressive increase in liver fat accumulation, even in individuals without NAFLD, which alters vitamin D storage, metabolism, and release of vitamin D-binding proteins (DBPs) in circulation needed to facilitate vitamin D distribution throughout the tissues since its lipophilic nature (^1^). The second step of vitamin D activation occurs through the activity of the renal enzyme 1α-hydroxylase (CYP27B1) which converts 25(OH) vitamin D into 1,25-dihydroxyvitamin D [1,25(OH)_2_ vitamin D] (also named calcitriol). Even in the absence of a clinically diagnosed form of chronic kidney disease (CKD), renal function progressively declines in the elderly impairing the activation of vitamin D metabolites (^11^). The impairment of re nal vitamin D metabolism results from a decline in CYP27B1 activity and a progressive loss of nephrons and reduced renal perfusion leading to a decline in glomerular filtration rate (GFR) by approximately 1 mL/min per year after the age of 40 years (^4^,^11^).

Different drug medications commonly used in older populations, such as corticosteroids, anticonvulsants and proton pump inhibitors (PPIs) can contribute to vitamin D deficiency in these subjects (^4^). Corticosteroids have profound detrimental effects on vitamin D metabolism, calcium homeostasis, and musculoskeletal health. Their long-term use promotes vitamin D degradation, impairs calcium absorption, and disrupts bone turnover significantly increasing the risk of osteoporosis, fragility fractures and sarcopenia, particularly in older adults (^12^-^14^). Corticosteroids negatively impact on vitamin D metabolism leading to upregulation of CYP24A1, the enzyme responsible for converting both 25(OH) vitamin D and 1,25(OH)_2_ vitamin D into inactive metabolites. In addition, corticosteroids suppress renal CYP27B1, the enzyme responsible for converting 25(OH) vitamin D into its biologically active form. Corticosteroids can also impair gastrointestinal absorption of vitamin D and calcium through gut microbiota alterations and intestinal barrier dysfunction (^15^). Anticonvulsants, including phenytoin, phenobarbital, and carbamazepine, significantly interfere with vitamin D metabolism by inducing hepatic cytochrome P450 enzymes, in particular CYP24A1 and CYP3A4, increasing the catabolism of both 25(OH) vitamin D and 1,25(OH)_2_ vitamin D (^16^). Patients taking antiepileptic drugs had up to a 30% lower serum 25(OH) vitamin D concentration compared to non-users predisposing to progressive bone loss (^17^,^18^), and long-term use of enzyme-inducing antiepileptic drugs is strongly associated with decreased bone mineral density (BMD) and increased risk of fragility fractures (^19^). PPIs are widely prescribed for gastroesophageal reflux disease (GERD) and peptic ulcer disease. Long-term use of PPIs has been demonstrated to impair the absorption of calcium and vitamin D by altering gastric pH (^20^). Indeed, these compounds inhibit hydrogen-potassium ATPase in gastric parietal cells leading to hypochlorhydria which reduces the food solubilization and the intestinal absorption of fat-soluble vitamins, including vitamin D (^21^), and long-term PPI therapy has been linked to an increased risk of osteoporotic fractures (^22^).

### Epidemiology of vitamin D deficiency in the elderly

Vitamin D deficiency is a widespread public health issue worldwide with older population as one of the most affected by this detrimental condition linked to age-related cutaneous changes, reduced sun exposure, inadequate dietary intake, and progressive impairment of liver and kidney functions (^23^-^25^). In Italy, between 60% and 80% of older subjects are characterized by suboptimal serum 25(OH) vitamin D levels (<50 nmol/L/20 ng/mL), with circulating levels decreasing in winter especially in northern regions. Among institutionalized elderly individuals, vitamin D deficiency issue is even more severe, with up to 90% experiencing severe deficient vitamin D status (<30 nmol/L/12 ng/mL). In the United States, NHANES data (2001-2018) indicate that 17.2% of women and 16.8% of men older than 80 years present circulating 25(OH) vitamin D concentrations <50 nmol/L/20 ng/mL, while 2.4% of women and 2.1% of men present levels below <25 nmol/L/10 ng/mL (^26^). The prevalence of subjects presenting 25(OH) vitamin D levels below 25-50 nmol/L/10-20 ng/mL is notably higher during winter months, affecting 28.9% of the population as compared to 17.0% in summer. Although the vitamin D deficiency issue is widely recognized to mainly affect populations living in geographic regions with limited sun exposure, an impaired vitamin D status highly characterizes also those living in tropical regions, especially in the elderly. In a recent crosssectional study including 212 community dwellers aged ≥80 years in Sao Paulo, Brazil (Lat 23.5 oS), vitamin D deficiency (defined with circulating 25(OH) vitamin D levels <20 ng/mL) and severe vitamin D deficiency (<10 ng/mL) were observed in the 56% and in the 13% of the cohort, respectively (^27^). *In vitro* model data also demonstrated an impaired cutaneous vitamin D3 synthesis during the winter months in the tropics (^28^), as confirmed by the pronounced seasonality observed in serum circulating 25(OH) vitamin D concentrations in the population living in these spe cific regions (^29^).

Based on the multiple risk factors for vitamin D deficiency in older adults, public health initiatives should consider screening for deficiency in highrisk groups, improving dietary intake through food fortification and promoting safe sun exposure and supplementation, as also recently stated in the updated guidelines of the Endocrine Society recommending supplementation in the elderly even without specific vitamin D status assessments (^30^). Evidence from Finland supports the effectiveness of food fortification in reducing vitamin D deficiency rates (^31^). After the generalized fortification of dairy products and fats in 2003, the prevalence of vitamin D deficiency in Finnish older population dropped from 12% to 0.6% between 2000 and 2011, with a mean serum 25(OH) vitamin D level increasing from 48 nmol/L to 65 nmol/L.

### Vitamin D deficiency in the elderly: definition and diagnosis

The accurate diagnosis of vitamin D deficiency is essential to prevent its detrimental impact in the elderly. Circulating serum 25(OH) vitamin D is widely recognized as the most reliable and useful biochemical marker of vitamin D status, reflecting both endogenous synthesis, dietary intake and supplementation (^5^). However, definition of the optimal serum 25(OH) vitamin D levels remains of ongoing debate with recent guidelines and consensus recommendations refining the diagnostic thresholds based on individual risk factors and different clinical scenario.

Based on bone health, the 2011 report on dietary requirements for calcium and vitamin D from the Institute of Medicine (IOM) recommended the following diagnostic thresholds for vitamin D status (^32^): deficiency in case of 25(OH) vitamin D levels <12 ng/mL (<30 nmol/L); insufficiency in case of 25(OH) vitamin D levels 12-20 ng/mL (30-50 nmol/L); sufficiency in case of 25(OH) vitamin D levels ≥20 ng/mL (≥50 nmol/L). The 2011 Endocrine Society clinical practice guidelines, focused primarily on skeletal health and prevention of osteomalacia, established the following diagnostic thresholds for vitamin D status (^33^): severe deficiency in case of 25(OH) vitamin D levels <10 ng/mL (<25 nmol/L); deficiency in case of 25(OH) vitamin D levels 1020 ng/mL (25-50 nmol/L), insufficiency in case of 25(OH) vitamin D levels 21-29 ng/mL (52.5-72.5 nmol/L), sufficiency in case of 25(OH) vitamin D levels ≥30 ng/mL (≥75 nmol/L). Of particular note, in 2024, the Endocrine Society propose an updated clinical practice guideline for vitamin D management in general population for the prevention of extraskeletal diseases avoiding measurements of 25(OH) vitamin D levels in older subjects (^30^). The Endocrine Society no longer endorsed 25(OH) vitamin D level diagnostic thresholds to define vitamin D sufficiency, insufficiency, and deficiency status, even though this recommendation was specifically related to general population for the prevention of extra-skeletal diseases and not to those with skeletal disorders. Concerning to the general population aged 50 to 74 years, routine 25(OH) vitamin D testing and vitamin D supplementation beyond the recommended Dietary Reference Intake for this population (^34^) were not suggested. Moreover, also in the general population aged 75 years and older a routine testing for 25(OH) vitamin D levels was not suggested, and an empiric vitamin D supplementation was recommended due to its potential to reduce the risk of overall mortality (^30^). Indeed, a systematic review including 25 trials with a total of 49,879 participants assessing the effects of vitamin D supplementation on allcause mortality was performed, and meta-analysis suggested that vitamin D use, in most trials given as a daily dose of cholecalciferol either alone or combined with calcium, reduces mortality compared to placebo, with an estimated absolute effect size of 6 fewer deaths per 1,000 people with no differences according to risk of bias, gender, calcium coadministration, vitamin D dosage (high vs standard), setting (community, hospitalized, institutionalized) and vitamin D status (^30^).

The 2024 Consensus Statement of the 6th International Conference “Controversies in Vitamin D” highlighted how vitamin D assessment and supplementation need to be based on specific patients’ clinical risk factors and, differently by the 2024 Endocrine Society’s recommendations, the Consensus Statement proposes a treat-to-targeted approach ensuring an adequate supplementation when most beneficial and effective, especially in the elderly (^5^). The 2024 Consensus recommended that in frail and high-risk older adults vitamin D supplementation is prioritized over measurement, as its deficiency is highly prevalent in this population. In addition, serum 25(OH) vitamin D levels ≥30 ng/mL (≥75 nmol/L) are recommended in these individuals to prevent skeletal fragility, and monitoring 25(OH) vitamin D levels during supplementation is recommended to ensure adequate vitamin D status and to modulate supplementation as appropriate.

With respect to vitamin D status biochemical assessment in older population, since circulating serum 25(OH) vitamin D levels also depend by DBPs, which serves as the primary transporter of vitamin D in circulation, fluctuations in DBP levels occurring in the elderly can impact on laboratory accuracy (^5^,^8^). Additional significant challenges in the vitamin D assessment are also due to variability in the laboratory measurement of serum circulating 25(OH) vitamin D which can be currently performed with different several commercial methodologies available in clinical practice including liquid chromatography-tandem mass spectrometry, immunoassays techniques and enzyme-linked immunosorbent assays, leading to potential inconsistencies between laboratories (^5^,^8^). Moreover, the presence of two serum 25(OH) vitamin D forms in circulation, 25(OH) vitamin D3 and 25(OH) vitamin D2, leads to additional difficulties in the biochemical assessment of the circulating total serum 25(OH) vitamin D concentration (^35^). Laboratory specimens available in clinical routine can differently detect and distinguish the two circulating forms potentially leading to underestimate or overestimate the vitamin D status in presence of substantial amounts of 25(OH) vitamin D2 (^5^,^8^). However, the specific concentrations of 25(OH) vitamin D2 in circulation are generally very low in most populations except in rare cases of highly use of vitamin D2 enriched food or specific supplements.

### Vitamin D deficiency in the elderly: impact on muscle, bone and adipose tissues

#### Muscle

Vitamin D is essential for muscle contraction, neuromuscular coordination, and muscle protein synthesis through its regulation of calciummediated signalling pathways (^36^). Several biological pathways support the effects of vitamin D on muscle. Vitamin D receptor (VDR) is essential in activating the intracellular signalling pathways relative to calcium metabolism and myoblast proliferation and differentiation, fundamental for preserving adequate muscle mass and strength (^36^). Vitamin D was demonstrated to enhance mitochondrial biogenesis increasing the expression of peroxisome proliferatoractivated receptor gamma coactivator 1-alpha and the upregulation of mitochondrial DNA copy number. In addition, it was proved to increase adenosine triphosphate production capacity and to improve oxidative function enhancing the activity of electron transport chain complexes (^37^,^38^). Conversely, vitamin D deficiency was proven to lead mitochondrial dysfunction, to decrease ATP and energy production, and to increase reactive oxygen species production and oxidative intracellular damage. Vitamin D deficiency was also linked to a dysregulation of myogenic regulatory factors, as the Notch signalling pathway, responsible for muscle progenitor cell differentiation and renewal (3638). Interestingly, vitamin D was also implicated in the muscle reparative process promoted by the gut microbiota which regulates muscle regeneration via RORγ+ Treg cells. These cells play a critical role in the homeostasis of extra-gut tissues and accumulate in the damaged muscle where can shield differentiating muscle stem cells from IL-17A (^39^-^41^). Vitamin D was recognized as a central actor in these processes by enhancing gut microbiota diversity, promoting T-cell plasticity, and by inducing the development of RORγt/FoxP3+ Tregs (^41^-^44^).

Clinical evidence also supports the role of vitamin D in preserving muscle mass, especially in older populations. A large study including 4,139 older adults assessed the relationships between vitamin D status and physical activity evaluated with different diagnostic tools, and reported that serum 25(OH) vitamin D levels and physical activity were linearly related to timed up-and-go test (TUG) performance and handgrip strength (^45^). A large meta-analysis including over 5,600 adult individuals with a mean age of 61.1 years showed a significant positive effect of vitamin D supplementation on muscle strength measured by grip strength, leg extension and quadriceps muscle strength. Importantly, the supplementation with vitamin D was most effective in improving muscle strength especially in subjects presenting 25(OH) vitamin D levels lower than 30 nmol/L (12 ng/mL) and who were 65 years of age or older (^46^). Similar findings were observed in another study showing an interactive effect between serum 25(OH) vitamin D and physical activity on functional tests and muscle strength (^47^). In a recent metaanalysis of 4 randomized controlled trials (RCTs) not specifically focused on older individuals, overall, a short-term treatment with moderate to high daily doses of vitamin D did not affect muscle health or quality of life even though a potential beneficial effect was present on muscle strength in severely obese subjects and on quality of life in those with vitamin D deficiency (^48^).

#### Bone

Vitamin D deficiency is associated with profound negative consequences for bone health, primarily by disrupting calcium-phosphorus homeostasis. Vitamin D promote sufficient intestinal calcium and phosphate absorption required for bone mineralization, and inadequate vitamin D status is known to impair this process contributing to osteomalacia and osteoporosis occurrence. In addition, vitamin D deficiency is recognized as a common cause of secondary hyperparathyroidism which exacerbates calcium mobilization from skeletal tissue to maintain serum calcium homeostasis (^49^). Chronic secondary hyperparathyroidism, typically observed in vitamin D-deficient individuals, is associated with increased bone resorption, cortical bone porosity and trabecular deterioration, increasing fragility fracture risk (^50^). In case of severe and prolonged vitamin D deficiency, the impaired bone mineralization caused by the lack of vitamin D leads to osteomalacia, a detrimental pathological condition characterized by an excess of unmineralized osteoid, skeletal deformities, and diffuse bone pain (^49^).

Clinical trials strongly support vitamin D supplementation as an effective strategy for treatment of osteoporosis and fracture prevention, especially when associated with calcium supplementation. Meta-analyses show that vitamin D supplementation, when combined with calcium, can reduce hip fractures by 16%-39% and non-vertebral fractures by 5%-26%, with the greatest benefits observed in high-risk elderly populations (^5^). The VITAL Study, a recent large RCT involving 25,800 adults, found that vitamin D3 supplementation, at doses of 2,000 international unit (IU) daily, without calcium, did not significantly reduce fracture risk over 5.3 years (^51^). Importantly, the participants enrolled in this trial were characterized by relatively high baseline 25(OH) vitamin D levels (mean levels of 30.7 ng/mL), with very few having severe deficiency (<12 ng/mL) which is the group of patients that might have benefited more from supplementation, potentially explaining the null results of the study. Additionally, different evidence suggest that vitamin D deficiency may also reduce the response to anti-osteoporotic therapies, such as bisphosphonates, potentially limiting their effectiveness in preventing bone loss and fragility fractures, as well as to predispose to secondary endocrine-driven osteopathies (^52^-^55^).

#### Adipose tissue

Vitamin D deficiency is well-known to be associated with obesity and related comorbidities, and adipose tissue is the main storage site for vitamin D and its metabolites. Indeed, vitamin D is a fat-soluble steroid hormone and its volumetric dilution into adipose tissue mass when increased is recognized as one of the most plausible explanations for the relationship between vitamin D deficiency and obesity (^56^,^57^). Experimental studies suggest that obesity is associated with decreased expression of specific genes that regulate the metabolism of vitamin D by altering synthesis of the enzymes CYP2R1 and CYP27B1 (^58^). On the contrary, an upregulation of CYP24A1 activity which promotes vitamin D catabolism has been suggested in experimental models (^59^).

Several other pathophysiological mechanisms have been proposed to explain the recognized association between vitamin D deficiency and obesity in humans (^60^-^62^). Patients with obesity tend to spend less time in outdoor activities with limited skin exposure to sunlight. Lower dietary intake of vitamin D, impaired hepatic 25-hydroxylation (^63^), impaired hydroxylation in adipose tissue, and alterations in VDRs (^60^-^62^) are additional factors. Obese patients are typically characterized by lower levels of 25(OH) vitamin D, which are inversely correlated with body mass index (BMI) and adiposity (^64^,^65^). The prevalence of vitamin D deficiency is reported to be 35% higher in individuals with obesity than in normal weight individuals (^64^). Moreover, obese patients often require larger amounts of vitamin D supplementation than their normal-weight counterparts. A recent meta-analysis showed that, after administration of equal doses of vitamin D, 25(OH) vitamin D levels were lower by about 15.2 ng/mL (38 nmol/L) compared with eutrophic individuals, with doses ranging from 4,0006,000 to 40,000-60,000 IU weekly (^66^).

On the other hand, a causal role for low serum 25(OH) vitamin D in body mass accumulation has also been suggested (^67^). Indeed, it was demonstrated that adipose tissue itself is a direct target of vitamin D which can influences its distribution, metabolic and endocrine functions (^68^). The VDR is present in the pre-adipocytes and adipocytes in both visceral and subcutaneous adipose tissue (^69^) and serves as the mechanistic mediator of these properties. *In vitro* studies on mouse adipocytes showed that calcitriol, the active form of vitamin D, increases basal and stimulated lipolysis and decreases lipogenesis (^70^) resulting in a catabolic reduction in adipocyte number and size by decreasing lipid and triglycerides accumulation. Conversely, increased parathyroid hormone secretion secondary to low 25(OH) vitamin D levels is associated with a raise of intracellular calcium in adipocytes stimulating lipogenesis and weight gain. On the other hand, with respect to energy expenditure in murine models, the knockout for VDR and CYP27B1 leads to a lean phenotype and less adipose tissue than wild type mice promoting resistance to dietinduced obesity (^71^,^72^). It was also demonstrated that CYP27B1 knockout mice presented lower leptin levels and consumed significantly more food than their wild type counterparts (^73^,^74^), and targeted expression of human vitamin D receptor in adipocytes reduced lipolysis, fatty acid beta-oxidation and induced obesity in mice (^75^).

Based on these findings, it has been suggested that vitamin D supplementation may have a role in obesity and associated metabolic disorders. However, the results of the main RCTs exploring the effects of vitamin D supplementation on body fat mass and body weight are still inconclusive (^76^), even if significant effects in reducing the risk of diabetes in high-risk populations and in positively modulating glucose metabolism in general population were consistently reported by several large studies (^77^-^81^).

### Vitamin D deficiency in the elderly: key-role in osteosarcopenic obesity

**Figure 1:**
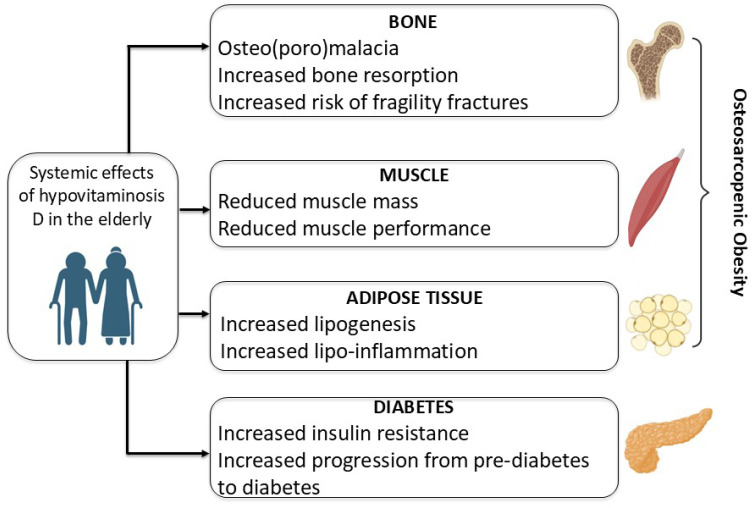
Systemic effects of hypovitaminosis D in the elderly.

Based on the findings described above, emerging evidence highlights the role of vitamin D deficiency in osteosarcopenic obesity, a pathological condition defined by the concomitant presence of osteoporosis, sarcopenia, and obesity, which can typically affect the older population (**Figure 1**) (^82^,^83^). Ostesarcopenic obesity is now recognized as a specific clinical entity with several metabolic alterations which occur in the skeletal muscle of individuals with obesity that may negatively impact muscle mass and function, and bone health (^82^-^86^). This pathological condition is often underrecognized, despite being associated with poor metabolic and functional outcomes (^87^). Epidemiological studies highlight that osteosarcopenic obesity has a prevalence ranging from 5% to 37% in older adults, depending on sex, ethnicity, and diagnostic criteria, with higher rates observed especially in the elderly and in individuals with metabolic syndrome (^85^,^86^). The coexistence of alterations in bone, muscle, and adipose tissue is demonstrated to exacerbate physical decline, insulin resistance and glucose metabolism impairment, and chronic systemic inflammatory processes, and to increase the risk of falls, fractures, daily impairment and mortality on the elderly (^82^). Inflammation and oxidative stress exert catabolic effects on skeletal muscle and bone loss, and fat accumulation with fatty muscle infiltration (myosteatosis) results in lipotoxicity and alterations in muscle stem cells determining a shift towards adipocyte differentiation (^88^).

In addition to the muscle damage and loss of strength, vitamin D deficiency was also implicated in promoting neuromuscular dysfunction and cognitive impairment. Consistent evidence supports that vitamin D plays a crucial role in brain health with its deficiency being associated with cognitive decline and an increased risk of neurodegenerative diseases (^89^). Several studies indicate that subjects with lower serum 25(OH) vitamin D concentrations are characterized by worse cognitive performances and by a higher prevalence of dementia (^90^,^91^). Clinical and preclinical researches suggest that vitamin D exerts its beneficial effects on brain through multiple pathways including the modulation of neuroinflammation, reduction of oxidative stress and enhancement of amyloid-beta clearance (^90^-^93^). The negative impact of vitamin D deficiency on cognitive function of older individuals further exacerbates fragility fracture susceptibility and falls (^4^). In individuals with osteosarcopenic obesity, the excess in visceral and intramuscular fat contributes to the reduced muscle quality and the impaired bone remodelling. Concomitantly, the increased vitamin D sequestration in the adipose tissue significantly reduces its circulating bioavailability, worsening bone loss and muscle weakness (^94^).

Beyond its direct effects on skeletal and muscle function, vitamin D is also known to regulate the bonemuscle crosstalk, modulating key endocrine mediators such as osteocalcin, sclerostin, VEGF, IGF-1, and myostatin, all of which are essential for bone integrity and muscle trophism (^36^,^82^).

A multicenter, controlled, double-blind, parallelgroup trial that included 380 sarcopenic older individuals, divided into an active group receiving vitamin D and protein nutritional supplementation or isocaloric control products only, showed that the intervention was effective in improving postprandial muscle protein synthesis, appendicular skeletal muscle mass, and lower-extremity function measured using the chair stand test (^95^).

Falls are a common and severe health issue among older adults, especially in those with osteosarcopenic obesity, leading to significant morbidity and mortality. Sarcopenia and osteoporosis have been directly linked to an increased susceptibility to falls, fractures, and mortality in multiple studies (^96^). Recent large RCTs found no significant reduction in fall risk with high-dose vitamin D supplementation in the elderly, however, the fall assessments in these trials were not rigorous (^97^). On the contrary, large interventional studies suggest a U-shaped relationship between vitamin D status and fall risk with optimal 25(OH) vitamin D levels for minimizing risk of falls ranging between 20-40 ng/mL, or as high as 60 ng/mL, with risk increasing at both lower and higher levels (^98^-^101^).

The high risk of fractures and falls associated with osteosarcopenic obesity in the elderly requires a comprehensive prevention strategy also including non-medical compounds as recently suggested in combining vitamin D supplementation and mechanical hip protectors (^102^). Indeed, besides the positive effect of vitamin D on skeletal and muscle health, hip protectors have been proposed as an adjunctive useful strategy to reduce fracture risk in frail older individuals. Although the compliance with these mechanical compounds remains a challenge, the technique advancement with next-generation airbag-based hip protectors is promising in improving adherence and fracture prevention outcomes (^102^). Thus, a multifaceted therapeutical intervention combining vitamin D supplementation, structured resistance training, and targeted nutrition may optimize muscle mass, bone density, and metabolic function in individuals with osteosarcopenic obesity (**Figure 1**).

### Vitamin D deficiency in the elderly: why, when and how to supplement?

The 2024 Endocrine Society guidelines have recently recommended the use of vitamin D supplementation in all adults older than 75 years, regardless of baseline vitamin D status (^30^). This approach was supported by suggesting that routine supplementation may be more cost-effective than 25(OH) vitamin D measurement testing given the high prevalence of vitamin D deficiency and its association with overall increased mortality in this population. Fortified foods and specific vitamin D supplements are recommended, with daily dosing preferred over high-dose intermittent regimens to require stable vitamin D serum levels and reduce adverse effects (^30^). The 2024 Consensus Statement recommended a treat-to-target supplementation strategy highlighting individual risk assessment rather than the same therapeutical approach for every subject (^5^). While most of clinical trials have yielded inconsistent findings about the efficacy of vitamin D supplementation for different extra-skeletal outcomes, Consensus recommendations highlighted the importance in characterizing high-risk groups as older individuals with osteoporosis, sarcopenia, obesity, CKD, or malabsorption syndromes, for whom maintaining serum 25(OH)D levels above 30-40 ng/ mL may potentially provide significant musculoskeletal and systemic benefits as reported in the post-hoc analyses of the major RCTs. The Consensus Statement supports selective vitamin D assessments also in this population to avoid unnecessary supplementation in those with sufficient levels and to stratify vitamin D deficiency severity identifying the more tailored therapeutical approach to adopt (^5^).

Vitamin D supplementation is available in different forms with distinct characteristics suitable for different specific clinical settings. Cholecalciferol (vitamin D3) is generally the preferred option for supplementation as it is characterized by greater bioavailability, a longer half-life, and a stronger effect in raising and maintaining adequate serum 25(OH) vitamin D levels as compared to ergocalciferol (vitamin D2) (^5^). However, in different specific clinical conditions an alternative approach may be required. For patients with severe vitamin D deficiency, malabsorptive syndromes, obesity, or liver impairment, calcifediol may represent a more effective therapeutical strategy (^5^,^8^). Differently to cholecalciferol, calcifediol bypasses hepatic hydroxylation leading to a faster correction of circulating 25(OH) vitamin D levels. In this regard, promising therapeutic strategies recently propose the use of biofortified foods directly with 25(OH) vitamin D3 also (^103^). In murine models, it was reported that biofortifying 25(OH) vitamin D3 in egg yolk effectively raises serum 25(OH) vitamin D concentrations than normal or high-fat diet. Other evidence from supplementation studies with hens showed that only dietary 25(OH) vitamin D3, and not vitamin D3 supplementation, resulted in a pronounced increase of 25(OH) vitamin D3 concentration in the eggs (^104^). Recently, the European Food Safety Authority concluded that the novel food calcifediol monohydrate proposed for use in food supplements in humans can be considered as a bioavailable and safe source of vitamin D under the proposed conditions of use and amounts, i.e. up to 10 µg/day for children ≥ 11 years old and adults, including pregnant and lactating women, and up to 5 µg/day for children 3-10 years of age (^105^). Thus, the efficacy and utility of potential dietary sources of 25(OH) vitamin D is worthy to be further investigated.

For individuals with moderate to severe CKD, inactive forms of vitamin D supplementation cannot be sufficient as the renal hydroxylation process is impaired. In these cases, the use of active vitamin D metabolites, such as calcitriol, or analogs like alfacalcidol, is necessary (^5^,^8^). However, active analogs are also characterized by a very short halflife and narrow therapeutic ranges with higher risk to induce hypercalcemia and vascular calcification, particularly in fragile older individuals (^5^,^8^).

In older adults, the 2024 Endocrine Society guidelines recommended that a routine daily vitamin D supplementation of 800-2,000 IU is generally sufficient (^30^,^32^-^34^), although it is wellknown that individuals with severe deficiency, obesity, chronic illnesses, or those on medications that affect vitamin D metabolism may require doses up to 4,000 IU per day (^5^,^8^). Even though the tolerable upper intake of vitamin D recommended by the National Academy of Medicine is set at 4,000 IU daily, when necessary, the use of higher doses may be considered given the demonstrated safety without specific severe concerns (^5^,^8^). The Calgary Vitamin D study showed that the safety profile of vitamin D supplementation, used in a cohort of healthy adults aged 55 to 70 years with serum 25(OH) vitamin D levels ranging from 12 to 50 ng/mL, was similar for doses of 400, 4,000, and 10,000 IU daily, and hypercalciuria, which occurred more frequently with higher doses, was mostly rare, mild, and transient (^106^).

Maintaining serum 25(OH) vitamin D levels above 20 ng/mL (50 nmol/L) is considered adequate for bone health and fracture prevention, while levels above 30 ng/mL may be needed in those with osteoporosis or calcium-phosphorus metabolism disorders (^5^). Although some studies explored the efficacy of highdose intermittent supplementations (*e.g.*, monthly or annual boluses of 50,000-300,000 IU), these therapeutic strategies were associated to potential increased risk of falls (^5^,^97^-^100^,^107^,^108^).

Finally, for individuals at higher risk of vitamin D deficiency, 25(OH) vitamin D levels monitoring can be recommended with a biochemical assessment 2-3 months after starting supplementation allowing clinicians to evaluate the patient’s response and adjust dosing, if necessary, due difficulty of certain high-risk groups in maintaining adequate vitamin D status is compromised by the underlying conditions (^5^).

### Vitamin D deficiency and COVID-19: two modern pandemics highly affecting the elderly

**Figure 2:**
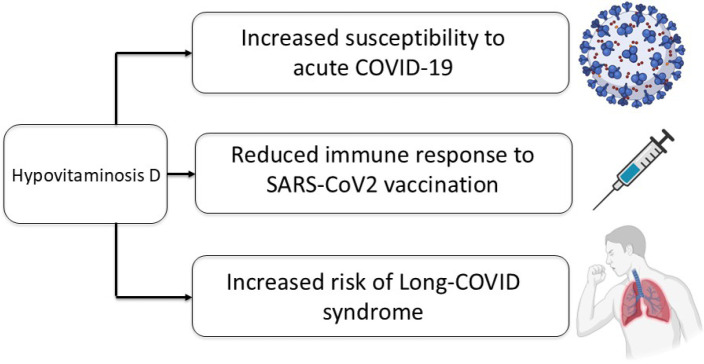
Impact of hypovitaminosis D in COVID-19 pandemic.

The knowledge about extra-skeletal effects of vitamin D was improved by the recent observations consistently associating vitamin D deficiency with the occurrence of worse outcomes in the COVID-19 pandemic, especially in older populations (^109^-^113^) (**Figure 2**).

Vitamin D is well-demonstrated to influence innate and adaptive immunity, supporting antimicrobial and antiviral immune responses (^1^,^5^,^114^). Several retrospective case-control studies, meta-analyses, as well as prospective studies with low-risk of biases, consistently revealed inverse associations between hypovitaminosis D and the risk of severe COVID-19 (^115^-^117^). In older patients affected by COVID-19, vitamin D deficiency was associated with a more severe lung involvement, longer disease duration and mortality risk (^118^). In addition, older individuals with vitamin D deficiency and critically ill COVID-19 were characterized by a high risk of delirium as compared to those with normal vitamin D status (^119^). The association between vitamin D status and COVID-19 outcomes in patients older than 60 years was assessed in a recent systematic review including 11 studies and reporting that those with vitamin D deficiency had worse clinical outcomes such as mortality, oxygen therapy and invasive mechanical ventilation requirement (^120^). The authors also showed that patients supplemented with vitamin D were characterized by better outcomes as compared to the non-supplemented group (^120^). In the COvid19 and VITamin d TRIAL (COVIT-TRIAL) study, a single oral high-dose of cholecalciferol administered at diagnosis improves the 14-day overall survival in adults older than 65 years as compared to the standard-dose cholecalciferol (^121^). These results are in accordance with the previous findings reported in different large meta-analyses, not only focused on older individuals, assessing the impact of vitamin D supplementation in COVID-19 patients and showing benefits mostly in reducing mortality and intensive care unit (ICU) admission rates (^122^,^123^). Even if most of the studies included in these meta-analyses specifically investigated the impact of supplementation with cholecalciferol form, promising data were also shown in studies assessing the efficacy of treatments with calcifediol demonstrating its benefits in reducing the risk for severe COVID-19, ICU admission and mortality (^124^-^132^). Given the lesser sequestration in adipose tissue, the shorter half-life, and the potential faster elicitable increase in circulating serum 25(OH) vitamin D concentrations obtained with calcifediol when compared to cholecalciferol, these data may be relevant for future investigations in this specific clinical setting, especially when treating severely obese patients or those with malabsorptive conditions as typically occurs also during acute systemic illness.

Interestingly, the relationships between vitamin D deficiency and adipose tissue and glucose metabolism were also observed in this specific clinical context. In a single center cohort-study, lower 25(OH) vitamin D levels were associated with high blood glucose levels and higher BMI in COVID-19 patients predicting a more severe disease (^133^). In particular, patients presenting both hyperglycemia and hypovitaminosis D, and those presenting hyperglycemia or hypovitaminosis D, were markedly characterized by worse outcomes as compared to those with normal glucose and 25(OH) vitamin D levels. Since vitamin D deficiency also characterizes diabetic patients with retinopathy (^134^), it was hypothesized that lower 25(OH) vitamin D levels could worsen the predisposition of patients with diabetes to the microvascular systemic damage typical of COVID-19. Accordingly, patients presenting both overweight and hypovitaminosis D, and those presenting overweight or hypovitaminosis D, were markedly characterized by worse outcomes as compared to those with normal BMI and 25(OH) vitamin D levels (^133^). These data supported the importance of a potential synergistic negative impact of hypovitaminosis D and adipose and glucose alterations as unfavorable prognostic factors in patients with acute COVID-19.

Besides the impact on acute COVID-19, the extraskeletal effects of this hormone in vitamin D were recently implicated also in influencing the post-acute disease recovery and Long COVID syndrome. Long COVID is a novel multisystemic syndrome involving multiple tissues and leading to neurocognitive, cardiorespiratory, constitutional, and musculoskeletal sequelae not explained by other medical diagnoses and potentially attributed only to the previous infection and continuing for more than 12 weeks after recovery (^135^). Several pathophysiological mechanisms have been proposed for Long COVID including viral persistence, hyperinflammatory state, immunological changes, and microbiota alterations (^136^), with an increasing risk observed in individuals with comorbidities such as obesity, glucometabolic and endocrine dysfunctions, and frailty (^137^-^142^). A potential role of vitamin D in the post-acute disease recovery of patients previously affected by COVID-19 was hypothesized on the basis of its multisystemic effects in influencing musculoskeletal health and function, in reducing nonspecific musculoskeletal pain, myalgia, and arthralgia (^143^,^144^), in influencing neurocognitive functions and disorders (^145^,^146^) and, also, in promoting respiratory recovery after pneumonia (^147^,^148^). In a single center cohort-study, lower 25(OH) vitamin D levels, evaluated at follow-up visits scheduled six months after hospital discharge, were observed in subjects with Long COVID than those without (^149^). Long COVID was diagnosed using the National Institute for Health and Care Excellence (NICE) guidelines with a multidisciplinary evaluation performed 6 months after hospital discharge (^135^). Regarding the affected health areas assessed, lower 25(OH) vitamin D levels were observed in particular in those with neurocognitive symptoms than those without. In addition, negative correlations between 25(OH) vitamin D and glucose levels at follow-up were also reported, reinforcing this association previously observed in patients with acute COVID-19 and in general population. This finding was proposed to represent an additional mechanism implicated in the Long COVID syndrome since in general population hypovitaminosis D was demonstrated to increase the risk of diabetes (^5^,^30^,^81^), and this latter was associated with a higher occurrence of Long COVID (^149^-^151^).

## CONCLUSION

The last decades, growing evidence highlights a marked association between vitamin D deficiency and different adverse health effects, especially in highrisk groups as the older population. These individuals are particularly susceptible to vitamin D deficiency on a multifactorial basis, and to the occurrence of its detrimental consequences. In the elderly, a progressive age-related impairment of bone, muscle and adipose health can typically occur, and these processes can be further worsened by the lack of an adequate vitamin D status. Vitamin D deficiency has been linked to impaired muscle function, bone deterioration and metabolic negative consequences (**Figure 1**). Thus, vitamin D deficiency appears to contribute to frailty in the elderly through multifaced mechanisms. This promotes the occurrence of an osteosarcopenic obese phenotype in older individuals exacerbating their systemic complications and overall morbidity and mortality. Given the complex interplay between vitamin D and bone, muscle and adipose tissues, the emerging problem of vitamin D deficiency in the elderly requires a well-structured approach including a prompt diagnosis and screening based on individualized criteria, a personalized therapeutic strategy with treat-to-target supplementation approaches, and a structured followup monitoring to optimize both safety and efficacy, enhancing the overall benefits of vitamin D on muscle, bone and adipose tissues, and ultimately improving the quality of life of this vulnerable older population.

## Data Availability

datasets related to this article will be available upon request to the corresponding author.
